# Current Standards and Perspectives in Proteomics for Cardiac Amyloidosis

**DOI:** 10.1021/acs.analchem.5c06139

**Published:** 2026-03-04

**Authors:** Francesco Greco, Ellen McPhail, Diana Canetti, Ahmad Masri, Yu Fu Ferrari Chen, Luca Menichetti, Alberto Aimo, Maria Franzini, Lidia Ciccone, Angela Pucci, Vincenzo Castiglione, Giuseppe Limongelli, Marco Merlo, Gianfranco Sinagra, Cristina Basso, Marianna Fontana, Julian D. Gillmore, Liam Andrew McDonnell, Michele Emdin, Giampaolo Merlini, Giuseppe Vergaro

**Affiliations:** † 366975 Fondazione Toscana Gabriele Monasterio, Pisa 56124, Italy; ‡ Fondazione Pisana per la Scienza ONLUS, San Giuliano Terme 56017, Italy; § Department of Laboratory Medicine and Pathology, Mayo Clinic, Rochester, Minnesota 55902 United States; ∥ Centre for Amyloidosis, Division of Medicine, Royal Free Campus, 4919 University College London, London NW3 2PF, U.K.; ⊥ Division of Cardiology, 6684 Oregon Health & Science University, Portland, Oregon 97239, United States; # 19005 Scuola Superiore Sant’Anna, Health Science Interdisciplinary Center, Pisa 56127, Italy; ∇ CNR-IFC, 117072 National Research Council Institute of Clinical Physiology, Pisa 56124, Italy; ○ Pathology Division, 9257 University Hospital of Pisa, Pisa 56124, Italy; ◆ Department of Pharmacy, 9310 University of Pisa, Pisa 56126, Italy; ¶ Department of Translational Medical Sciences, Inherited and Rare Cardiovascular Diseases, 18994 University of Campania ‘Luigi Vanvitelli’, Naples 80131, Italy; △ Cardiothoracovascular Department, Azienda Sanitaria Universitaria Giuliano Isontina, 9247 University of Trieste, Trieste 34149, Italy; ▲ Department of Cardiac, Thoracic, Vascular Sciences and Public Health, 9308 University of Padua, Padua 35128, Italy; ▽ Department of Molecular Medicine, 19001 University of Pavia, Pavia 27100, Italy

## Abstract

Amyloidosis is a heterogeneous group of protein misfolding disorders characterized by the deposition of amyloid fibrils in tissues, which causes progressive organ dysfunction. Among the various forms, cardiac involvement by transthyretin (ATTR) and immunoglobulin light chain (AL) amyloidosis is particularly significant, as it is the main determinant of prognosis and treatment. In clinical practice, tissue biopsy remains crucial for diagnosing amyloidosis. Amyloid histological typing can be performed using antibody-based methods such as immunohistochemistry and immunoelectron microscopy. Mass spectrometry-based bottom-up proteomics has emerged as a powerful alternative, offering precise identification and quantification of amyloidogenic proteins. Despite being regarded as an effective technique for amyloid typing, the application of proteomics remains limited to specialized centers due to its technical complexity and lack of standardization in clinical workflows. This paper describes the current use of proteomics in patients with cardiac amyloidosis, based on the experience of referral centers in the USA and Europe, to provide guidance on improving the technique’s reliability and to identify standard practices, challenges, and gaps in the clinical application of amyloidosis typing.

## Graphical abstract

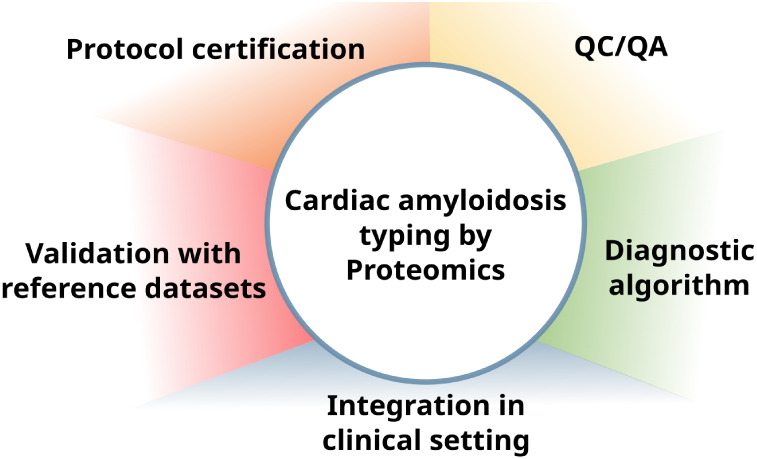

## Introduction

Cardiac amyloidosis (CA) is a protein misfolding disease caused by the accumulation of fibrillated proteins (often transthyretin (ATTR) or immunoglobulin light chains (AL)) in the cardiac muscle, leading to organ dysfunction. Protein deposit characterization is crucial since the protein type is the main determinant of prognosis and treatment. Tissue biopsy is often needed to characterize the composition of the amyloid deposit.^1^ Despite amyloid characterization having traditionally been performed by immunohistochemistry, mass spectrometry-based bottom-up proteomics has emerged as a powerful tool for identifying and quantifying the proteins present within amyloid deposits. Although there is room for improvement in terms of reliability, optimized mass spectrometry-based proteomics protocols are considered one of the most powerful tools in amyloid typing.^2,3^ Amyloidosis typing by proteomics was first reported more than 15 years ago;^4–6^ today it is not standard in clinical practice and is limited to specialized centers. Here, we report the experience of different centers in the USA (Mayo Clinic, Rochester, MN, USA) and in Europe (UCL Center for Amyloidosis, London, UK; Fondazione Toscana Gabriele Monasterio, Pisa, Italy; Amyloidosis Research and Treatment Center, IRCCS Policlinico San Matteo, Pavia, Italy) that use proteomics for amyloidosis typing. This paper aims to summarize the conclusions of a dedicated workshop held in Pisa on November 8, 2024, some of them also discussed within the European Proteomics Amyloidosis Network (EPAN) community in Kiel.^7^ The goal of the paper is to discuss improvements in technique reliability by proposing standardization, validation, and certification strategies, with the ultimate goal of promoting the clinical integration of proteomics in the management of CA.

## Amyloid Typing by MS-Based Proteomics

Liquid chromatography–mass spectrometry-based proteomics analysis of amyloid deposits for amyloid subtyping was first reported in 2008.^5–8^ Currently, there is no standard for amyloid typing, and different protocols have been developed for sample processing and analysis, depending on specimen type and instrumental availability.^7^

CA typing is most typically performed using endomyocardial biopsies (EMBs) or surrogate site (e.g., periumbilical fat) aspirates. The most common pipeline involves laser capture microdissection-based (LCM) enrichment of amyloid deposits from congo red (CR)-stained formalin-fixed, paraffin-embedded (FFPE) endomyocardial or periumbilical fat biopsies^5,7,9–11^ (Figure 1A). The analysis of periumbilical fat aspirates is performed in some laboratories via sample homogenization and processing without prior fixation,^6,12,13^ due to the patchy and/or scanty nature of the amyloid deposits and the risk of loss of amyloid tissue during processing (Figure 1B).

**Figure 1:**
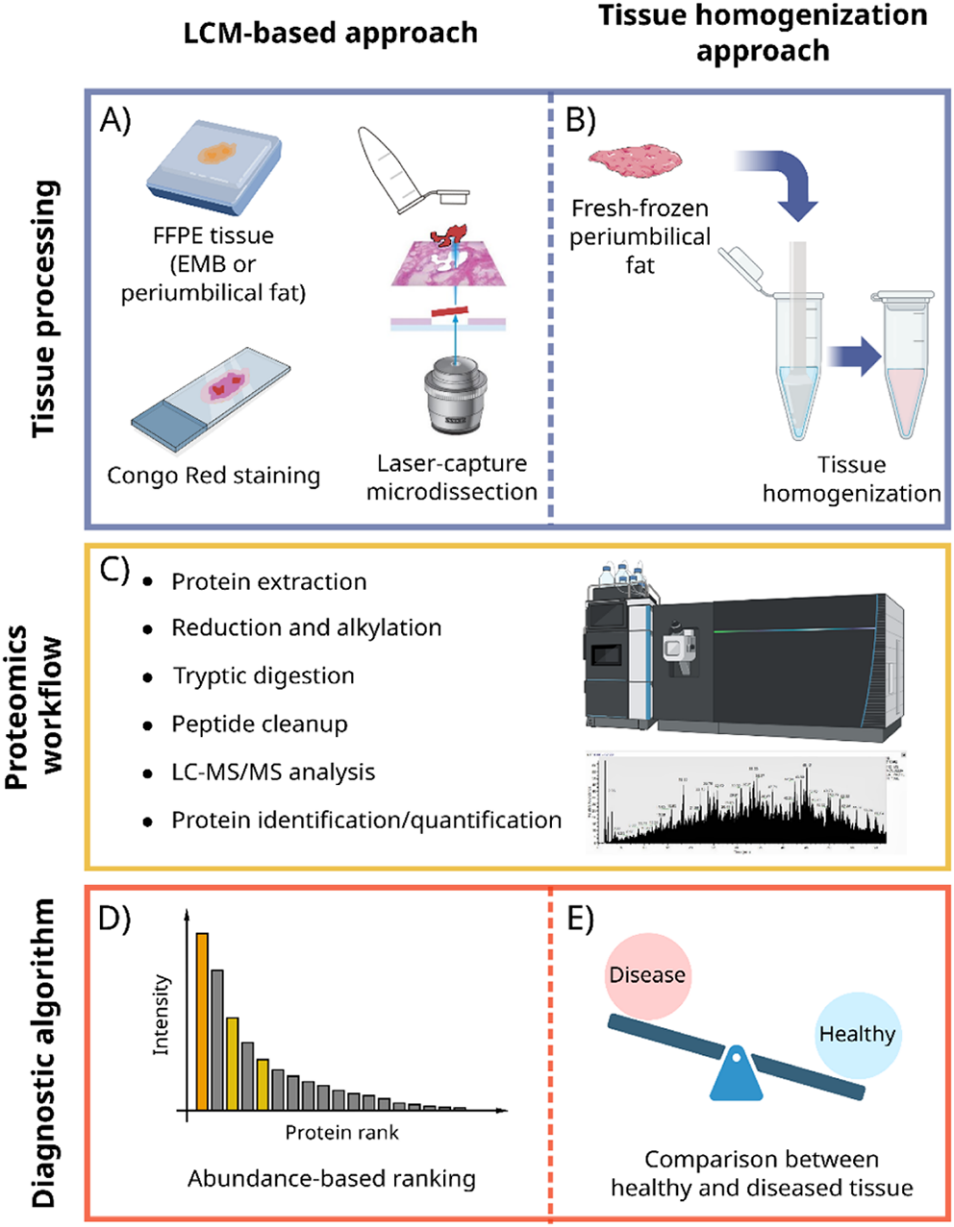
Schematic of the LCM-based and tissue homogenization-based approaches to amyloidosis typing. A) FFPE endomyocardial biopsy (EMB) or periumbilical fat is enriched in amyloid protein by LCM. B) Fresh-frozen perimumbilical fat is homogenized as a whole. C) In both approaches, the sample is processed and digested, and peptides are analyzed by LC-MS/MS. D) For LCM-enriched samples, the most abundant/intense amyloidogenic protein is recognized as the main constituent of the deposit. E) For the tissue homogenization approach, the protein profile of the sample is compared to the protein profile of healthy tissue to detect amyloidogenic proteins.

Proteins are extracted from FFPE samples by heating in the presence of surfactants (15 min to overnight).^4–6,13–16^ After cysteine reduction and alkylation,^13,14^ proteins are digested by trypsin at 37 °C overnight.^5,14,15^ Peptides produced by proteolytic digestion are purified by C18 solid-phase extraction columns, vacuum-dried, and resuspended for injection into the LC-MS/MS system (Figure 1C). Peptides are separated by LC and analyzed by tandem mass spectrometry. To date, most amyloidosis typing experiments using LC-MS/MS methods have been data-dependent analyses (DDA),^4,5,10,12,14,15,17^ i.e., the masses and intensities of the peptides are measured in an MS1 spectrum, and abundant peptides are then isolated and subjected to MS/MS to obtain structurally informative fragments. The resulting MS/MS spectra are matched to an *in-silico* generated database for peptide identification. Recently, data-independent analysis (DIA) methods, which are based on the subdivision of the scanning range into larger isolation windows, have been proposed for amyloid typing.^18,19^ In DIA, fragmentation spectra are generated from multiple precursors and matched with a DDA-generated library for peptide identification and quantification. Targeted approaches, based on the monitoring of AL-κ, AL-λ, and ATTR peptide fragments, have also been developed for amyloidosis typing.^13^ Search databases can be expanded to include peptides containing known amyloidogenic variants to detect the mutation directly from LC-MS/MS data.^20,21^

Currently, there is no standardized approach to obtaining a diagnosis from MS data. The most common approach, used especially with LCM-enriched samples, is based on the assumption that the most abundant amyloidogenic protein detected in the amyloid-enriched sample is the main protein in the deposit^4,5^ (Figure 1D). Protein abundance has been estimated using Mascot score^22–25^ or spectral counts,^10,17,26–31^ in a few cases normalized to the total spectral counts of all amyloidogenic proteins (α-value).^9,12,32^ Protein intensities have also been used to estimate protein abundances.^19,33^ In the case of whole tissue digestion, the protein profile of homogenized tissue is compared to the protein profile of healthy tissue to detect amyloidogenic proteins^12^ (Figure 1E). The presence of amyloid signature proteins (apolipoprotein E, apolipoprotein A-IV, and serum amyloid P-component), proteins known to coprecipitate with the amyloidogenic protein, in conjunction with the most abundant amyloid precursor protein, can provide support for amyloid typing in homogenized/whole fat aspirate specimens.^10^ The presence of this amyloid signature^10,14^ has been explored as a marker of amyloidosis and has demonstrated superior diagnostic performance compared to CR in diagnosing amyloidosis in subcutaneous fat, despite the absence of a full clinical validation.^10^

## Role of Proteomics in the Diagnostic Workup of Cardiac Amyloidosis

Mass spectrometry-based proteomics plays a crucial role in the diagnostic workup of CA by enabling precise amyloid typing, which is essential for prognosis and for guiding targeted therapy. Traditional antibody-based methods, such as immunohistochemistry (IHC) and immunofluorescence, have limitations, including cross-reactivity and nonspecific background staining, resulting in difficulties in distinguishing amyloid subtypes. Cross-reactivity of the amyloid deposit to more than one antibody is due to the fibrillar form of the protein exposing different epitopes from its native form, against which the antibody is usually developed. Moreover, in AL, the fibril is usually composed of variable regions of the immunoglobulin, while antibodies are specific for the constant chain. Antibodies against the fibrillated form of amyloid proteins have been developed to improve specificity, but they are not largely used in clinical practice.^34,35^ Some of these limitations could be mitigated by using larger antibody panels together with rigorous and standardized staining protocols. Nonetheless, for EMB, the size of the tissue sample is often too small to provide the number of sections needed for a large antibody panel, and periumbilical fat exhibits an amyloid deposit in less than 50% of ATTR cases.^36^ Despite these disadvantages, the relatively low cost of the analysis and the already established integration of IHC in clinical practice make antibody-based techniques one of the most useful amyloid-typing methods in clinics. Immunoelectron microscopy (IEM) is a reliable and direct technique to find and type interstitial amyloid fibrils with good sensibility and specificity. Its limitations include cost, access to transmission electron microscopy with dedicated personnel, and the availability of antibody panels.

Liquid chromatography-tandem mass spectrometry (LC-MS/MS) overcomes these challenges by directly analyzing the protein composition of amyloid deposits, allowing for the highly specific and sensitive identification of amyloid precursor proteins such as TTR, light chains, and rare amyloidogenic proteins from a single tissue section. Moreover, LC-MS/MS can enable differentiation between wild-type and hereditary forms of TTR amyloidosis,^21^ which is critical for genetic counseling and treatment planning.^37^ The turnaround time of proteomics-based amyloid typing can vary between different centers but usually spans from 2 to 3 days in dedicated laboratories to 1–2 weeks, depending on the availability of personnel and instrumentation.

The integration of proteomics into clinical practice has revolutionized CA diagnosis by minimizing misclassification, reducing the risk of inappropriate therapies (e.g., unnecessary chemotherapy in non-AL cases), and enabling personalized treatment strategies. The CA diagnostic pathway can include many technologies, spanning from clinical imaging (cardiac magnetic resonance and bone scintigraphy) to tissue biopsy characterization. Proteomics has become an important part of the diagnostic pathway, able to reinforce diagnoses in the case of concordance with the other techniques and to reduce the number of inconclusive results by complementing antibody-based techniques. In the rare cases in which proteomics and antibody-based assays are not concordant, it would be advisable to repeat IHC and proteomics analysis on consecutive tissue sections. It is important to note that for CA, the final clinical diagnosis results from the comparison and interpretation of all the data available for the patient, and thus, there should be no absolute preference for proteomics or IHC, but both results should be interpreted case by case in the clinical context.

Despite its advantages, LC-MS/MS remains less widely available than IHC due to the need for specialized equipment and expertise. Nevertheless, its adoption in referral centers is improving diagnostic accuracy and advancing precision medicine in CA.

## Do We Need Proteomics Protocol Standardization for Amyloidosis Typing?

The proteomics workflows for amyloidosis typing were developed independently by proteomics research laboratories prior to their application in the clinical setting. Accordingly, each clinical center uses a different protocol, and there is poor harmonization in terms of sample preparation steps, reagents, instrumentation, data acquisition strategies, and data analysis. Each protocol reflects the expertise and available infrastructure of each specific center. Despite methodological and technological differences, many reported methods have been compared to antibody-based methods^12^ or clinical diagnoses,^15,33,38^ confirming their efficacy regardless of rigorous overall method standardization.

A mass spectrometry-based protocol for clinical applications should provide reliable and consistent typing results to prevent misdiagnosis. To comply with these requirements, there are different protocol standardization/validation options.

One possibility would be the adoption of the same fixed and standardized protocol across all of the different centers, complemented by extensively described standard operating procedures. This rigid standardization would enable more reproducible proteomics measurements and a direct comparison of results from different clinical centers. This strict standardization is likely not needed, since amyloid typing is already performed successfully by different groups using different methods and instrumentation. Moreover, the imposition of a defined protocol may raise an obstacle to the clinical adoption of proteomics-based amyloid typing in laboratories that are not equipped with the instrumentation defined by the standardized protocol. Another disadvantage would be that a standardized protocol would be less likely to incorporate upgrades from novel research.

A second option would be a method-oriented validation, consisting of testing, for each laboratory-specific protocol, some crucial steps (e.g., tissue fixation time, minimum amount of microdissected tissue) following shared guidelines. The advantage would be that each laboratory could use its own protocol as long as critical parameters of crucial steps are under control. Moreover, the definition of thresholds for critical parameters is useful to set the ranges of applicability of the protocol. On the other hand, this validation strategy is more focused on technical aspects rather than on diagnosis. Moreover, it is difficult to define common crucial steps for protocols that are very different (e.g., laser capture microdissection-based protocol, whole tissue digestion approach).

A third option to ensure the quality and reliability of amyloidosis typing using proteomics would involve the validation of each proteomics protocol by using an established validation cohort. According to this approach, the focus would be shifted from the proteomics process to the resulting diagnosis. The only requirement would concern accurate amyloidosis typing and would be tested via the consistent and correct diagnosis of a known data set to which the analyst was blind. The disadvantage would be that this validation would be highly dependent on the quality of the validation cohort, which would require interlaboratory efforts to be curated and maintained.

The EPAN community is moving away from strict standardization of a single proteomics-based amyloid typing protocol, and it is leaning toward method-oriented validation of laboratory-specific protocols flanked with large-scale interlaboratory validation using shared validation cohorts, despite an official document still lacking.

## Proteomics Protocol Validation and Benchmarking: Reference Samples

The effort towards an interlaboratory validation of a proteomics-based typing protocol starts with the establishment and characterization of a set of reference samples. A comprehensive characterization of the reference samples should include multiple approaches (Figure 2), including histological confirmation of CR positivity and birefringence, immunohistochemistry/immunofluorescence, immunoelectron microscopy, and mass spectrometry.^39^ The results of these techniques should be in agreement and coherent with the clinical record and follow-up of the donor patient. A round-robin LC-MS/MS study of reference samples, involving established amyloidosis proteomics centers, would provide a benchmark for new centers seeking to introduce LC-MS/MS into their amyloidosis typing workflow.

**Figure 2:**
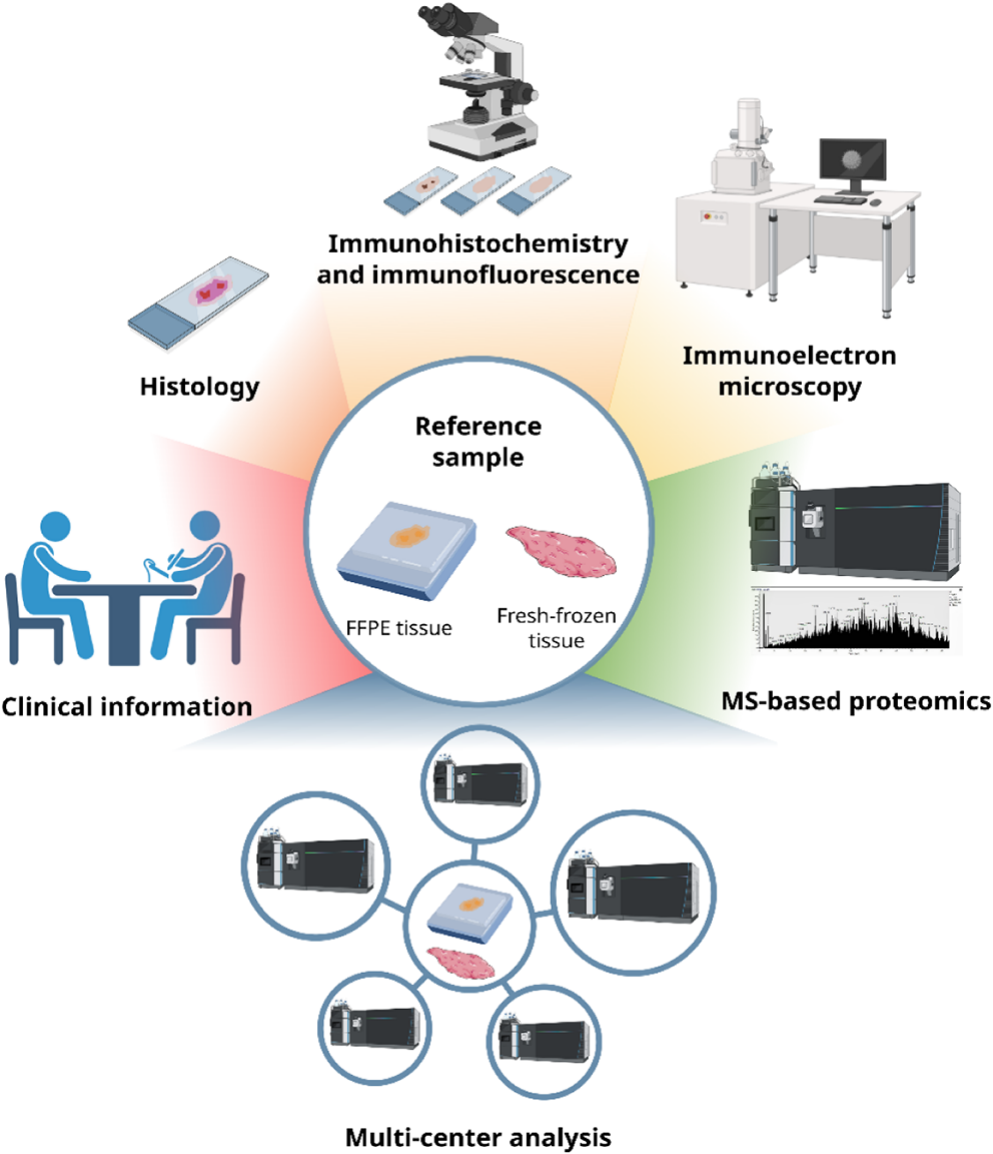
Schematic representation of the possible strategies to correctly assign an amyloidosis diagnosis and amyloidosis type to a reference sample for MS-based proteomics validation. Integration of clinical information, histology, immunohistochemistry/immunofluorescence, immunoelectron microscopy, and MS-based proteomics can all contribute to amyloidosis diagnosis. Multicenter analysis allows for a correct characterization of reference samples.

A representative and meaningful validation set should include all types of CA samples expected in clinical practice in terms of tissue types, amyloid proteins, amount of material, and involvement of more than one amyloid type. Setting up and maintaining a large validation set takes a lot of effort and sample material, since sections from the same samples have to be used for both characterization and actual method validation by multiple laboratories. The size of a validation set should take into consideration case representation, the effort needed to establish the cohort, and the amount of tissue in reference samples.

The most common biopsy sites in CA are EMB and umbilical fat, the latter being positive for amyloid in less than 50% of ATTR cases.^36^ Especially for EMBs, the amount of available tissue is limited, and only a few analyses can be run on each sample, making them unsuitable for interlaboratory validation cohorts. Specimens from amyloidomas (localized amyloid masses in the absence of systemic amyloidosis) are usually abundant and could thus provide material for reference samples.^40^ However, amyloidomas are only caused by either light chain deposition or, rarely, by serum amyloid A, and are therefore not representative of many amyloidogenic proteins.^41,42^ Despite being subjected to longer fixation times, autoptic specimens or explanted hearts with amyloidosis could be an abundant source of reference tissue and could be used to validate MS-based proteomics protocols. Formalin fixation creates a polymer network between the tissue’s constituent proteins, and longer fixation times reduce protein extraction yield and thereby have an effect on protein detection.^43,44^ Nonetheless, fixation issues have never been reported for amyloidosis typing by proteomics.

Amyloidosis samples for a validation cohort should be characterized by many techniques. Nonetheless, the validation cohort should not include only samples for which there is full agreement, since it would bias the validation data set toward “easy” typing cases. For this reason, it would be recommended to include some challenging cases, for which one of the techniques (e.g., antibody-based or proteomics) provided a discordant result, while all the others were coherent with the patient’s clinical record and follow-up. The validation cohort might also include cases of highly characterized dual pathology to ensure the capability of the tested method to recognize this condition or, at least, flag the sample for further analysis.

The establishment of a reference validation cohort that fulfills all the discussed requirements is challenging. In practice, a central institution would be needed to provide the physical storage space for reference samples, to curate the database, and to organize validation rounds across institutions (e.g., by mailing CR-stained sections). Annual meetings could be platforms to organize the validation rounds, discuss results, and share validated protocols and best practices, while experienced centers could organize training courses to share methodologies and support the setup of the protocol in other centers. In the clinical setting, FFPE and fresh samples are usually managed by the pathology department. The support of the pathology department of amyloid-typing institutions would be critical in the establishment of a meaningful validation cohort, since pathologists could already provide histological evaluation and antibody-based diagnosis, while the institution that followed the donor patient could provide clinical information for each reference sample (e.g., clinical imaging, patient history, and genetics).

## Guidance for Amyloidosis Typing Diagnostic Algorithm

Raw LC-MS/MS data from amyloid typing protocols need to be processed to obtain a clinical diagnosis. Proteins are identified and quantified using Mascot score, spectral counts, or protein abundances. Currently, there is no consensus on which protein quantification method to use. It could be argued that Mascot score is a measure of the identification probability,^45^ which is only indirectly linked with protein abundance. Spectral counts have been largely used in the past as a semiquantitative method, despite being dependent on the duration of the dynamic exclusion from fragmentation^46,47^ and on protein length.^47^ Both identification score and spectrum counts have been shown to perform worse than other intensity-based quantification methods for discriminating differences in protein abundance.^47,48^ CA typing differs from the more established clinical application of quantitative proteomics, namely, differential expression analysis, which compares the measured intensity of the same protein in different samples; in CA typing, the comparison is between different proteins in a single sample. In this case, protein intensities can be biased by the number of tryptic peptides of each protein and their susceptibility to ionization. Other types of protein intensities normalized by protein length, such as IBAQ (Intensity-Based Absolute Quantification), might be more appropriate.^19^ A rigorous comparison between quantification approaches is still missing, and currently, there is no consensus on the optimal quantification strategy. Nonetheless, all of the previously mentioned approaches have been successfully applied to amyloid typing.

Protein quantifications are processed by a diagnostic algorithm to obtain a diagnosis. Diagnostic algorithms are generally based on the identification of the most abundant amyloidogenic protein in the sample, but more complex procedures involving normalization^12,32^ or decision trees^16^ have been described.

Diagnostic algorithms should include internal controls to avoid interference from circulating proteins arising from extensive blood contamination or from blood vessel/serum components present in the tissue alongside the amyloid deposit. Over 95% of all cardiac amyloidosis cases are of either AL or ATTR type.^11^ Since all systemic AL amyloid patients and up to 40% of ATTR patients have a concomitant monoclonal protein,^49^ up to half of all cardiac amyloid patients will have a monoclonal protein in their serum. It is essential to distinguish the presence of serum contamination by a background monoclonal immunoglobulin in an amyloid specimen from true AL amyloidosis. It is recommended to evaluate potential blood contamination by comparing the abundance of immunoglobulin proteins with serum albumin, hemoglobin chains, or fibrinogen beta and gamma chains. An algorithm to discriminate fibrinogen amyloid from circulating fibrinogen “contaminating” the biopsy specimen has been proposed and tested on renal biopsies.^22^

The diagnostic algorithm should include objective criteria for the definition of conclusive/inconclusive results. Proteins that coprecipitate in the amyloid deposit, the so-called amyloid signature proteins (Serum amyloid protein P, Apo A-IV, and ApoE^50^), should be used as an internal quality control metric to confirm amyloid deposition. The detection of an amyloid protein without the simultaneous detection of the amyloid signature proteins could be used to flag inconclusive results.

The diagnostic algorithm should ideally be able to flag samples of double amyloidosis, which can occur in three different scenarios. In the first, the patient has two different anatomic sites involved by two different amyloid types.^29,51,52^ A possible example is a diabetic patient with insulin amyloidosis in abdominal fat and ATTRwt amyloid in the heart. The second scenario is a patient with two different amyloid types in two different anatomic compartments within the same biopsy specimen. For example, in their report of dual amyloid patients, Sidiqi et al. described a 79-year-old man with CA whose endomyocardial biopsy showed ATTR involving interstitial amyloid deposits and AL-lambda involving vascular amyloid deposits. The patient was subsequently diagnosed with renal AL-lambda forming vascular amyloid deposits.^29^ Identification of different amyloid types in different anatomic compartments can be achieved by careful laser microdissection and separate processing of amyloid deposits from the individual compartments. In the third scenario, a single anatomic compartment within a single anatomic site is involved by two different amyloid types.^52^ This is the most challenging dual amyloid scenario to diagnose, especially if one of the two amyloid types is AL, which is frequently the case. In these situations, it is imperative to distinguish background contamination by serum immunoglobulins from true AL amyloidosis to enable optimal patient management. Currently, there is no established proteomics scoring algorithm that can reliably detect double amyloidosis cases.

## Certification of the Proteomics Protocol

The clinical application of proteomics requires certification to comply with diagnostic standards, as results inform therapeutic decisions.^53^ The ISO 9001 certification and ISO 15189 accreditation ensure compliance with quality standards with respect to quality controls, laboratory management, reagent tracking, instrument maintenance, and risk assessment. Certification/accreditation of the proteomics workflow involves the codification of the protocol into operating procedures to ensure the repeatability of sample processing and data analysis. Registration of samples and reports must be included, as well as registers and documentation of instrumental nonconformity. Risk assessment is required to evaluate the possible pitfalls of the procedure and their probability to plan counteractions.

Different kinds of quality controls are needed in a certified protocol.^53^ Quality controls are needed to evaluate instrumental performance and the reagents. Validation samples need to be run periodically to maintain the diagnostic accuracy of the protocol. Adhering to regulatory standards will ensure data reliability, thus supporting clinical decision-making in amyloidosis diagnosis (Table 1).

**Table 1:** Quality Control Samples Analysis Implemented to Obtain and Maintain the College of American Pathologists/Clinical Laboratory Improvement Amendments (USA)^a^

	Sample type	Frequency	Outcome
**Process-level** **QC**	Reference FFPE blocks (AL-κ, AL-λ, ATTR, and AA)	One FFPE block for each sample batch	Correct diagnosis
**LC control**	Reference FFPE blocks (AL-κ, AL-λ, ATTR, and AA)	All blocks processed weekly	28 performance metrics extracted by MassQC^54^ (width of chromatographic peaks, mass measurement error, number of identified peptides). Parameters must fall within standard operating limits.
**MS/MS** **control**	Reference FFPE blocks (AL-κ, AL-λ, ATTR, and AA)	All blocks processed weekly	

a QC: Quality Control. LC: Liquid chromatography. MS/MS: tandem mass spectrometry.

## Integration between the Proteomics Laboratory and the Clinical Team

The demand for proteomics-based typing of amyloidosis stems from a clinical necessity. Two primary models have emerged to define the relationship between the proteomics laboratory and the clinical team. These can be broadly described as the “external service provider model”, where the laboratory functions as an independent entity offering diagnostic services, and the “internal multidisciplinary team model”, where the laboratory is integrated into the clinical team, enabling close collaboration and streamlined workflows.

The “external service provider model” is a high-throughput and high-expertise technological platform that relies largely on proteomics for tissue-based diagnosis, with CR-positive samples being processed without immunohistochemistry or immunoelectron microscopy confirmation. This approach relies entirely on proteomics typing and is implemented in centers that process a large number of samples, many of which may be processed as a service for external clinics.^55^ In this setting, an internal clinical evaluation of each case is not routinely performed, and the integration of the proteomics data with the clinical data is performed by the external contracting center that submitted the service request. However, even in the external service provider model, the proteomic features are reviewed by a trained pathologist in the context of the morphology and CR staining (and clinical context, if possible) prior to rendering a diagnosis.^11^

In the second approach, proteomics-based amyloidosis typing is one element within a multidisciplinary diagnostic framework, which integrates proteomics with clinical, genetic, and imaging data (including results of other typing techniques such as immunohistochemistry and/or immunoelectron microscopy).

Both models are equally valid, and the choice between them realistically depends on the center’s capacity and the clinical context. In general, the “external service provider model” can be highly focused on proteomics techniques, processing a larger number of samples. The advantage of the “external service provider model” is that it provides access to proteomics-based amyloid typing for small centers that have no resources to implement a proteomics laboratory. This model centralizes the expensive equipment and expertise, optimizing resources. On the research side, access to large and homogeneous data sets allows for proteomics-based retrospective studies that are crucial to validate proteomics-based typing across a large number of patients.

The second approach is, in general, more focused on the integration of proteomics with other diagnostic techniques, and thus, the capacity in terms of the number of patients can be reduced. This model is more suitable for regions where already established proteomics laboratories can support local clinical centers by performing amyloid typing alongside other proteomics research. Importantly, the CA typing experiments benefit from the extensive and expensive efforts needed to ensure the LC-MS/MS is maintained in optimum working conditions and can benefit far more quickly from emerging proteomics methods/technologies. On the research side, these centers have access to a smaller but well-characterized number of patients, which allows them to investigate in detail the relationship between different diagnostic techniques.

## Perspective toward Integrated Diagnostic Pathways: Combining Proteomics and Clinical PET Imaging

In recent years, there has been growing interest in integrating molecular imaging modalities, such as positron emission tomography (PET), with proteomics data to enhance the diagnostic and prognostic accuracy of CA. PET imaging, using amyloid-binding tracers such as [18F] florbetapir—originally developed for Alzheimer’s disease—provides noninvasive, whole-body visualization of amyloid burden and distribution, offering functional and anatomical information that complements tissue-based proteomic characterization.^56^ Additionally, experimental tracers targeting the serum amyloid P component (SAP) or transthyretin (TTR) are under investigation, aiming to achieve subtype-specific PET imaging. Among these, the novel PET tracer [124I] Evuzamitide has emerged as a particularly promising candidate for cardiac amyloidosis, enabling high-resolution, quantitative imaging of amyloid deposits with a long physical half-life suitable for delayed imaging protocols.^57^ Early clinical data suggest its potential to sensitively detect cardiac involvement and systemic amyloid load, thus supporting both diagnosis and longitudinal monitoring.^58^

While proteomics enables precise amyloid typing at the molecular level, PET imaging can detect cardiac involvement early in the disease process, monitor treatment response, and assess extracardiac involvement in systemic amyloidosis. Integrating LC-MS/MS-based proteomics with PET imaging may provide synergistic insights, especially in cases of diagnostic ambiguity, inconclusive biopsy results, or suspected dual amyloid pathology. This multimodal approach has the potential to stratify patients more accurately, improve subtype-specific therapeutic decisions, and monitor disease progression or remission over time.

Moreover, PET imaging could assist in guiding biopsy site selection, particularly in patients with limited clinical symptoms or challenging anatomical access, thereby increasing the diagnostic yield of proteomic analysis. Future research and clinical protocols should aim to validate standardized workflows that integrate proteomic data and imaging of biomarkers. This convergence of technologies may ultimately facilitate precision medicine strategies in amyloidosis and support a comprehensive understanding of disease heterogeneity across molecular, structural, and functional dimensions.

## Amyloid Typing beyond LC/MS-MS

Amyloidosis typing is usually performed by either isolating the CR-positive areas by LCM or homogenizing the tissue. Proteins are extracted, digested, and analyzed by mass spectrometry. Spatial proteomics techniques, such as MALDI-MSI and DESI-MSI, can instead be used to create spatial maps of proteolytic peptides in tissue after *in situ* tryptic digestion. Recently, MALDI-MSI was proposed as an alternative to in-liquid proteomics workflows for tissue biopsies.^18,59^ Such MSI methodologies could reduce analysis time, remove the need for microdissection, and allow for the detection of amyloidogenic proteins from reduced amounts of sample, like in the case of thin perivascular deposition.^60^ To date, amyloid typing by MSI has proven challenging due to the reduced opportunities for protein denaturation *in situ* and thereby reduced digestion efficiency, the lack of a chromatographic peptide separation step, and the difficulties connected with peptide fragmentation in MALDI-MSI. Recent developments in instrumental sensitivity, combined with sample preparation have begun to allow amyloid typing by MSI.^59^ Spatial proteomics could also be important in the study of the changes of the tissue environment during amyloid deposition, by investigating spatial protein colocalization and changes in extracellular matrix composition. Furthermore, CA detection and typing by infrared spectroscopic imaging and artificial intelligence, which provide a nondestructive, rapid, and automated approach to CA diagnosis, hve recently been described.^61–63^

It is important to remember that establishing the correct amyloid type is essential for correct patient management, and the consequences of an incorrect diagnosis can be profound, if not fatal. Furthermore, 42 different human amyloid types have been described, including at least 9 different types in the heart. The current gold standard is LC-MS/MS, whose sensitivity and specificity are each close to 100% when enough material is present in the sample and in the absence of blood contamination. Using a very small amount of tissue, LC-MS/MS is capable of unambiguously identifying all of the amyloid types in a single assay. Newer methods must exceed these standards before they can be adopted for clinical use.

## Long-Term Strategy and Future Perspectives

Amyloid typing by mass spectrometry-based proteomics is already an established technique, but there remains room for improvement in terms of reliability and application in the clinical setting. It is true that CA typing by proteomics must involve experienced clinical centers to ensure the protocol is based on a large number of well-described cases, but it must also involve experts in the proteomics analytical method to ensure the protocol is analytically validated and that processes are implemented to ensure continued analytical rigor.

It is advisable to perform proteomics when IHC is inconclusive or an amyloid protein other than TTR or AL-lambda or kappa is detected, or if there is a discrepancy between the IHC result and clinical/imaging diagnosis. In fact, proteomics can complement IHC, providing a diagnosis in IHC inconclusive cases^16^ or, in some cases, correcting the IHC diagnosis.^15^ Both LCM and tissue homogenization-based protocols are effective and are usually applied to different sample types. Rather than the protocol, the crucial aspect of amyloid typing is process control.

Quality control and quality assurance (QC/QA) are critical for providing a reliable diagnosis. Overall protocol performance, as well as chromatography and MS/MS performance, should be monitored by specific controls. Protocol certification can provide guidance in the establishment of QC/QA procedures and in quality improvement, while method-oriented validation could provide the ranges of applicability of each protocol.

Effort should be put into building a well-defined validation cohort for amyloid typing that would allow for multicenter testing. Since the tissue in EMB and fat aspirates is usually limited, more abundant sources of reference material could be used, such as autoptic heart and local amyloidomas. Reference sets should be characterized by multiple techniques, including antibody-based IHC/IF and immunoelectron microscopy. Diagnosis should be consistent with the patient’s clinical record, including the presence of monoclonal proteins, bone scintigraphy, and genetic analysis.^1^ The successful typing of these samples would confirm that the method is suitable for amyloid typing and could be implemented for clinical applications.

Due to the significant effort involved in setting up and validating proteomics-based amyloid typing, and due to the multidisciplinary nature of the expertise involved in amyloidosis diagnosis and treatment, the most suitable healthcare managing system for amyloidosis is the hub-and-spoke model, with qualified referral centers involved in diagnosis linked to smaller local establishments. The number of “spokes” and the nature of the “hub” (e.g., a referral center specialized in amyloid typing only or a referral center that implements proteomics alongside other typing techniques), together with resource availability, will strongly influence whether it is more efficient to establish an “external service provider” or a “multidisciplinary diagnostic framework” model. The diagnostic pathway of cardiac amyloidosis may include multiple techniques such as cardiac magnetic resonance, bone scintigraphy, and genetics. For CA, the classical hub-and-spoke model would benefit from different levels of clinical care in the local establishments, while advanced diagnostic techniques such as proteomics and genetic analysis are implemented only in the hub, as already suggested by Italian statements on diagnostic and therapeutic pathways for CA.^64,65^

Aside from diagnostics, proteomics is a powerful technology with the potential to investigate the protein content of biological samples. We trust that proteomics will also be used to investigate the pathophysiology of the disease, its onset and progression, and the effect of fibril deposition on surrounding tissue.

## Conclusions

Amyloidosis typing is the first bottom–up proteomics application to be routinely used in clinical practice. Proteomics-based amyloidosis typing in CA has been a revolution in amyloidosis diagnosis, allowing for the reliable characterization of the amyloidogenic protein in the deposit. As various protocols for proteomics-based amyloid typing are being increasingly implemented in the clinical setting, a discussion of protocol definition, data analysis, validation, and certification is crucial for rigorous application.

The experience of specialized centers, experienced in applying proteomics technologies to CA typing, is fundamental to promote the use of proteomics and to provide guidance for the shift from research to clinical application.

## Abbreviations

ATTR: transthyretin amyloidosis
AL: light chain amyloidosis
CR: Congo Red
CA: cardiac amyloidosis
IHC: immunohistochemistry
IEM: immunoelectron microscopy
LC: liquid chromatography
MS/MS: tandem mass spectrometry
MALDI: matrix-assisted laser desorption/ionization
DESI: desorption electrospray ionization
MSI: mass spectrometry imaging
LCM: laser-capture microdissection
FFPE: formalin-fixed paraffin-embedded
DDA: data-dependent analysis
DIA: data-independent analysis
PET: positron emission tomography
SAP: serum amyloid P component
TTR: transthyretin
QC/QA: Quality control and quality assurance
